# Supplementary material for: Predicting visual function by interpreting a neuronal wiring diagram
Source: Nature. 2024 Oct 2;634(8032):113–23. doi: 10.1038/s41586-024-07953-5 (PMC11446822; doi:10.1038/s41586-024-07953-5)
Supplement: Supplementary file 6 — CRF and ERF predictions for individual TmY4 and TmY9 cells. Analogous to Supplementary Data 3, but for TmY target types. Shown are the top four monosynaptic pathways, the strongest pathway passing through each of the top ten intermediary types (ranking from Extended Data Fig. 7), and the trisynaptic pathway Tm1–TmY–Dm3–TmY (see the section entitled Prediction of spatial normalization). [file 41586_2024_7953_MOESM6_ESM.zip › DataS4/TmY9qΓèÑ/720575940632162631.pdf]

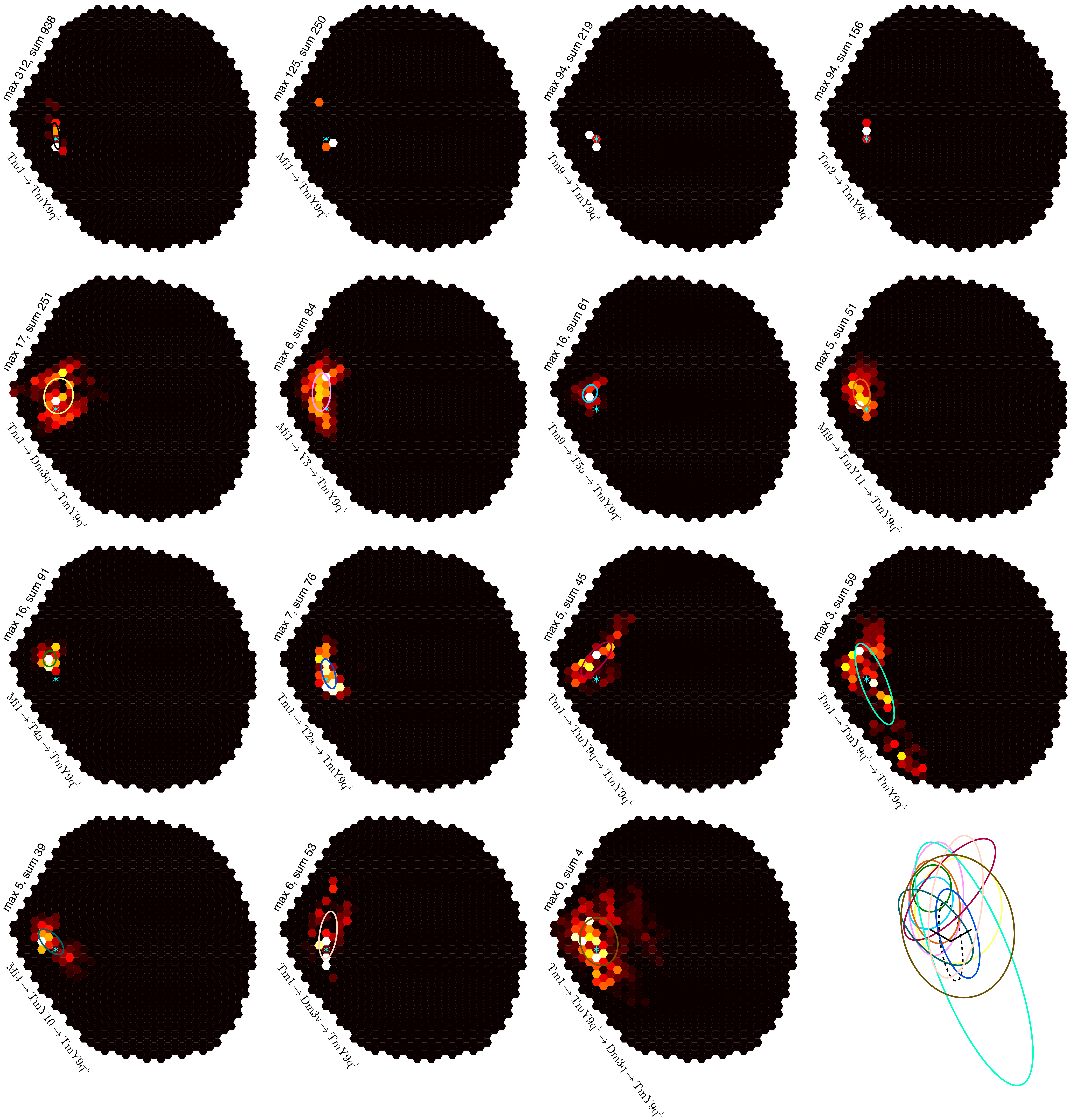
max 312, sum 938
Tm1 → TmY9q⊥
max 125, sum 250
Mi1 → TmY9q⊥
max 94, sum 219
Tm9 → TmY9q⊥
max 94, sum 156
Tm2 → TmY9q⊥
max 17, sum 251
Tm1 → Dm3q → TmY9q⊥
max 6, sum 84
Mi1 → Y3 → TmY9q⊥
max 16, sum 61
Tm9 → T5a → TmY9q⊥
max 5, sum 51
Mi9 → TmY11 → TmY9q⊥
max 16, sum 91
Mi1 → T4a → TmY9q⊥
max 7, sum 76
Tm1 → T2a → TmY9q⊥
max 5, sum 45
Tm1 → TmY9q → TmY9q⊥
max 3, sum 59
Tm1 → TmY9q⊥ → TmY9q⊥
max 5, sum 39
Mi4 → TmY10 → TmY9q⊥
max 6, sum 53
Tm1 → Dm3v → TmY9q⊥
max 0, sum 4
Tm1 → TmY9q⊥ → Dm3q → TmY9q⊥
